# Health-related quality of life from the perspective of children with severe specific language impairment

**DOI:** 10.1186/s12955-015-0326-1

**Published:** 2015-08-14

**Authors:** Kristy Nicola, Pauline Watter

**Affiliations:** The University of Queensland, Brisbane, QLD 4072 Australia

**Keywords:** Specific language impairment, Health-related quality of life, Communication disorders, Pediatric Quality of Life Inventory^TM^ 4.0 Generic Core, Quality of life

## Abstract

**Background:**

This study aimed to evaluate the feasibility and reliability of the Pediatric Quality of Life Inventory™ 4.0 Generic Core Scales (PedsQL™) for use by children with severe specific language impairment (SLI) and their parent, and to explore the health-related quality of life of children with severe SLI. We hypothesized that the PedsQL™ would be a suitable measure, and identify lower health-related quality of life compared to the healthy population sample, particularly in school and social functioning.

**Methods:**

Forty-three out of 61 children with severe SLI enrolled at a dedicated school from February 2010 until September 2011 agreed to participate. Children and parents completed the PedsQL™ separately with support as required.

**Results:**

The PedsQL™ proved to be suitable for this cohort. Children perceived themselves to be at risk of impaired social and physical functioning, rendering the total score below the population mean. Parents rated social and emotional functioning at risk of impairment, with the psychosocial and total summary score consequently below the population mean. Physical functioning had the largest child/parent difference, with children rating themselves below the cut-off score, and parents rating their children above the cut-off score.

**Conclusions:**

This measure can be used with this group. Our group of children with severe SLI reported lower health-related quality of life than the healthy population mean as perceived by both the child and the parent. Health professionals working with children who have SLI need to consider not only a child’s impairment, but also their wellbeing and participation by incorporating self- and proxy-reports into assessment in order to promote meaningful therapeutic outcomes that impact positively on a child’s life.

## Background

Childhood communication disorders can include speech and/or language problems to the extent that some children require special education intervention. Speech and/or language impairments that stem from no clear cause are considered to be primary, whereas impairments resulting from a developmental disability such as hearing impairment, are referred to as secondary. Approximately 7 % of school-aged children will unexpectedly fail to develop language, confirmed by performing poorly on tests of receptive and/or expressive language [1, 2]. Further investigations confirm that the language problems occur in the absence of not only cognitive impairment, but also any other condition that could explain the deficit. This presentation represents a primary language disorder called specific language impairment (SLI) [1, 2]. Emotional/behavioral issues [3], along with gross and fine motor comorbidities are not unusual in this cohort of children [4]. Consequently, SLI has both immediate and long-term influences on children’s lives, with many of these influences extending beyond the language domain often leading to academic underachievement [5], along with problems in developing and maintaining interpersonal relationships [6].

Health-related quality of life (HRQOL) is the perception of the impact an illness/injury, medical treatment and/or health care policy has on one’s life [7]. HRQOL is multidimensional and includes physical functioning, along with psychosocial dimensions of emotional, social and any related constructs [8]. HRQOL has been explored in many child populations, using either generic or condition-specific questionnaires. It is agreed that a child reporting his/her own perceptions is optimal, however in paediatric populations it may only be possible to explore a child’s HRQOL indirectly, via parents/guardians or health professional’s completion of a proxy-report [9]. Considering the array of challenges faced by children with SLI, this diagnosis has the potential to negatively affect every day functioning across various domains and consequently the HRQOL of these children. Yet little attention has been dedicated to the impact SLI has on a child’s HRQOL. Children 8–11 years old (*n* = 55) diagnosed with receptive-SLI (confirmed by language scores falling 1standard deviation (SD) below the mean) had different profiles when compared to norms, and scored significantly lower in speech and sleep domains of the 17D questionnaire [10]. Although these differences were not large enough to impact the overall HRQOL score, the results do provide preliminary evidence that even a mild presentation of SLI can influence HRQOL domains [10]. To date, no further studies have investigated the HRQOL perceptions from children with SLI and their parents.

Extending beyond a diagnosis of SLI, Markham et al. [11] presented the only other paper that reported the child’s perception of the impact speech and/or language problems have on his/her HRQOL. Markham et al. [11] used focus group interviews to discuss the perceptions of 29 children and young people aged 6.6–18.8 years with a broad range of speech and/or language problems, enrolled fulltime in either mainstream (*n* = 22), or special schools for children with communication needs (*n* = 7). This study reported that the speech and/or language difficulties of these children where perceived by the children to impact negatively on their HRQOL [11]. In addition, Markham et al. [11] reported credible evidence that children with speech and/or language difficulties can provide insight into their own HRQOL, and that common themes found in these discussions closely reflected domains of generic HRQOL measures [11]. These findings complemented a previous study conducted by Markham & Dean [12] where parents (*n* = 11) of children 2–9 years of age with speech and/or language difficulties, along with speech language pathologists (*n* = 12), and other professionals (*n* = 12) (such as educational staff), came together in focus group interviews to discuss their perceptions of the impact speech and language difficulties have on the HRQOL of predominately mainstreamed children [12].

A few additional studies have used proxy-reporting to explore the HRQOL of children with communication disorders. Parents of 3-year-old children with a language disorder completed the TNO-A21 Pre-school Children Quality of Life Questionnaire, which revealed that all children with language disorders, irrespective of the criteria used for identifying a language problem, had a lower HRQOL, discriminating 3-year-old children with language problems from those without [13]. In a follow-up study the parents who reported a persistent language disorder in their 8-year-old child, completed the Child Health Quality of Life Questionnaire - parent form 28 and rated their child’s general behavior, self-esteem, mental health, attitude towards schoolwork, emotional stability and psychosocial summary score all significantly lower than children without language disorders [14]. Therefore, emerging evidence suggests that mild speech and/or language disorders have the potential to impact negatively on a child’s HRQOL as perceived by the child or his/her proxy.

An explanation for the lack of attention devoted to this cohort of children, may be related to the challenges associated with selecting a suitable HRQOL measure.

A recent review explored the literature to identify the measures used for children with speech and language disorders [15]. Out of the four measures identified, three of these would not have been useful for the current study cohort, as two were designed for adult use, while another was for children younger than 6 years. The final measure, the Pediatric Speech and Language Quality of Life Scale, may be suitable for use with children with severe SLI, particularly when a condition-specific HRQOL measure for use with children with SLI is non-existent. However, this measure was not available at the time of data collection.

It is becoming accepted that SLI is not simply a language impairment, but is a heterogenic disorder where children may present with problems impacting cognitive and motor function. Indeed, condition-specific measures need to be specifically attuned to the particular ways in which communication disorders impact the HRQOL of children, while being sensitive and specific enough to identify small and important differences relevant to children with speech and language disorders [15]. However, it is equally important to gain a holistic understanding of the child’s HRQOL, particularly with conditions that have the potential to impact various HRQOL domains, and ideally allow for comparison against a range of paediatric conditions. Therefore, in order to explore the extent that the diverse problems experienced by children with severe SLI are impacting on the child’s perceived HRQOL a generic measure was sought. Most generic paediatric HRQOL measures have not yet been proven valid and/or reliable for use with children/adolescents with speech and language disorders [15], including children with SLI.

The Pediatric Quality of Life Inventory™ Version 4.0 Generic Core Scales (PedsQL™) [9] is one of the most commonly used generic measures for exploration of HRQOL in both healthy children and different cohorts of children with various diagnoses. However, this measure has yet to be explored for use with children with SLI [15]. The validity and reliability of the PedsQL™ is well established [8, 9] for both child self-report (from 5 years of age) [16] and proxy-report (from 2 years of age) [17]. This measure is particularly appealing for use with children with severe SLI due to the use of visual cues and developmentally appropriate language (consistent with the expected reading level of a child below the youngest target age of the particular questionnaire version) [9, 18]. It also allows comparison to be made amongst children with a variety of disabilities or impairments. Varni et al. [8, 16, 17] performs a consistent series of statistical analyses to explore initial feasibility, reliability and interpretation of results on the PedsQL™ to determine the suitability of the PedsQL™ for use with a particular cohort, for example children with developmental disorder [19]. These analyses completed by Varni et al. [8, 16, 17] have been published and supported for use in studies exploring HRQOL measures prior to Varni et al. [8, 16, 17]. The PedsQL™ has not been validated for use with children with speech and language disorders despite demonstrating sensitivity and responsiveness in other populations [15]. This study is a first step in addressing this gap by exploring the suitability of the PedsQL™ for use with children with severe SLI.

The HRQOL of children with severe SLI needs to be explored, as severe presentations require more understanding and support. Reporting children’s own perceived HRQOL will assist parents/guardians, professionals and policy makers to gain insight into the needs of this group. Further, it would assist service planning to holistically address identified issues to achieve optimal outcomes in children with a severe presentation. Therefore, this study aimed to examine the initial feasibility and reliability of the PedsQL™ in school-aged children with severe SLI and their parents. The method employed by Varni et al. [8, 16, 17] and colleagues [19] for confirming suitability of the PedsQL™ will be used in this study. If the measure is suitable, we further aim to explore and compare the HRQOL perceptions of children with severe SLI and their parents, to the normative population, to identify if these children, or gender and age subgroups of them, are at risk for impaired HRQOL. Given the previous findings, it is hypothesized that the PedsQL™ will be an appropriate measure for use with this cohort and will identify a high risk of impaired HRQOL compared to the healthy population mean, with the largest differences being in social and school functioning, regardless if reported by the child or his/her proxy.

## Methods

### Participants

In Queensland, Australia, one school provides educational and therapeutic support for children with such severe primary speech and/or language impairments that it prevents them from accessing curriculum in mainstream school. Acceptance into this school is through strict multidisciplinary screening by staff employed by the school. Children must meet the following criteria to be eligible for enrolment into the dedicated school: 1) perform at least 1.5SD below the mean on a psychometric test of general language ability, 2) have an overall standard score IQ >70 (mean = 100, SD = 15) performing better on the non-verbal test of IQ compared to language ability and 3) have confirmed failure of normal language development in the absence of other possible causes e.g. social-emotional disorders. In addition to the enrolment process, children who meet a prescribed strict protocol, where standardized assessment results fall at least 2SD below the test mean, have the potential to be screened by a state-wide verifier employed by The Department of Education, Training and Employment (DETE), (Government sector within Queensland Australia) to determine eligibility for enrolment into the speech-language impairment category within the Education Adjustment Program [20]. If a child is not eligible for inclusion in the speech-language impairment category, he/she may be considered for the intellectual disability or even the autism spectrum disorder category. This program is aimed to respond to the educational needs of students with disabilities by providing funding to help with the additional expenses required to support these students to access curriculum and participate in school life. Further information can be accessed on the DETE website [21].

For the purpose of this study, children were included if they met the following criteria: 1) 5–18 years old and enrolled at the school between February 2010 to September 2011, 2) verified only as speech-language impaired by DETE and 3) had SLI defined as receptive and/or expressive language deficits with or without speech impairment as confirmed via assessment file audit. Any children with secondary language impairment, speech impairment only, or with a diagnosis and/or categorized by DETE into the autistic spectrum disorder or intellectual disability categories were excluded. Therefore, the final group of children exhibited severe SLI with/without concomitant speech impairment, where expressive and/or receptive language proficiency scores fell greater than 2SD below the test mean.

The University of Queensland’s Medical Research Ethics Committee and the school’s Research Committee granted ethical approval to invite all children who met the criteria, and their parents to complete questionnaires. Teachers sent information and consent packages home with eligible children. Parents signed consent and completed the proxy-report at home, then returned them to the school. Parents were given the contact details of the principal researcher should they require any support in completing the questionnaire. After parent consent was gathered, researchers obtained the respective child’s assent and proceeded to administer the questionnaires. Children completed their questionnaires, separately from their parent, during school time in a quiet location. The questionnaires were interview-administered by research assistants for all children aged 5–7 years. Children aged 8 and older were encouraged to independently complete their questionnaires, however, most children required or preferred interviewer-administration. When children independently completed the questionnaires, a researcher was available to assist if required. As much time as necessary was allowed for children to complete the questionnaire. Given the nature of difficulties experienced by children with severe SLI, it was pre-empted that there may be general difficulties in administering a questionnaire to this cohort of children. It has been reported that it is possible to access the perspectives of adults with severe communication impairments if appropriate support is provided [15]. Therefore, a principal researcher and teacher aide were present to provide additional support if necessary, particularly in the form of using sign language to translate written text word for word to the children, a complementary communication that this school employs and in which the children were fluent.

### Instruments: Health-related Quality of Life Measure: The Pediatric Quality of Life Inventory™ Version 4.0 Generic Core Scales (PedsQL™) [9]

The PedsQL™ is a multidimensional 23-item measure that includes physical, emotional, social and school functioning scales. The PedsQL™ encompasses complementary child self-report and proxy-report that are both divided into multiple age versions. All versions are considered equivalent as the items are identical and differ only in developmentally appropriate language and tense. We used the following child self-report and equivalent proxy-report versions: 5–7 years (young child version), 8–12 years (child version), and 13–18 years (adolescent version). Individuals completing the measure must rate how much of a problem each item has been in the last month. A 5-point Likert scale is used for children aged 8 and over, while the measure is further simplified for children seven and under to a 3-point Likert scale that is anchored to a pictorial representation to assist younger children. Each item within a scale is reverse-scored and linearly transformed to a 0–100 scale. The mean of the transformed item scores is referred to as a scale score, where higher scores indicate better HRQOL and “at risk” scores can be identified as falling below the cut-off point. Scale scores can be used to develop various summary scores as follows: Physical Health Summary Score (the physical functioning scale score), Psychosocial Health Summary Score (the mean of emotional, social and school functioning scales), School Health Summary Score (the school functioning scale score) and Total Summary Score (the mean of all scales).

### Statistical analysis

Data were managed using SPSS [22]. The feasibility of the PedsQL™ as an outcome measure was explored by calculating the percentage of missing values for each item [8, 16, 17, 19, 23]. Cronbach’s alpha coefficient was used to explore the scale internal consistency reliability for both the self-report and proxy-report to ensure each scale’s reliability was above 0.70 to allow for group comparison, or 0.90 for analyzing individual patient scale scores [8, 16, 17, 19]. Analysis of child and proxy scores were completed separately [24]. To assist in identifying acceptability and sensitivity of this measure for this cohort the distribution of scores were explored by calculating the percentage of extreme range scores, that is the maximum possible score (ceiling effect = 100) and the minimum possible score (floor effect = 0) [19]. It has been proposed that younger children who find Likert scales difficult, may navigate to the ends of the response scales in order to reduce the responses to essentially a yes/no answer [18]. This is relevant to this cohort, as one could presume that should a child with SLI struggle with answering a question, he/she would similarly navigate to either end of the response scale, resulting in a ceiling or floor effect. Ceiling/floor effects within 1–15 % were considered acceptable [16, 17, 19, 23, 25]. Descriptive statistics were explored and the scale scores for children with severe SLI were compared to scores of healthy children, by applying the cut-off point scores (1SD below the healthy population mean) provided by Varni et al. [8] to identify if this cohort is at-risk for impaired HRQOL. To determine the difference between self and proxy raters a Mann Whitney U test for nonparametric analysis was used to compare the mean scores, because correlations have been identified as potentially being insufficient [24]. Similarly, the Mann Whitney U test was used to explore the effect of gender and age, with responses pooled as per the age ranges of the PedsQL™ questionnaires, namely 5–7 years, 8–12 years and 13+ years [9].

## Results

### Defining the study group

There were 99 children enrolled at the school, of whom 61 met the inclusion criteria of severe SLI with/without concomitant speech impairment. All 61 children and their parents were invited to participate in this study of which 43 (70 %) volunteered. The final group included 43 children with severe SLI and their parents. Since enrolment into this school requires children to meet strict criteria, non-participants were similar to the participants with regards to performance on standardized tests of language and cognitive ability, and therefore were not likely to differ in severity from participants. Descriptive statistics for the final study group can be found in Table 1.

**Table 1:** Descriptive statistics for study participants

Child characteristics			
Age range	Mean age	Males	Females
5–16 years	8.79 years	*n* = 35 (81 %)	*n* = 8 (19 %)
Proxy characteristics			
Proxy-reporter	Tertiary qualification	Unemployed	Employment classification^c^
Mother	*n* = 11 (32 %)^a^	*n* = 15 (36 %)^b^	Managers (3, 7.1 %)
*n* = 43 (100 %)			Professionals (29.3)
			Technicians & trades workers (1, 2.4 %)
			Community & personal service workers (4, 9.8 %)
			Clerical & administrative workers (4, 9.8 %)
			Labourer (1, 2.4 %)

^a^thirty-four mothers (34/43, 79.1 %) provided their educational level

^b^one mother (1/43, 2.3 %) did not provide her employment status

^c^as per the Australian and New Zealand Standard Classification of Occupations Major Group Classifications [31]

### Feasibility: missing item responses

The percent of missing items was 0.0 % for both child self-report and parent proxy-report.

### Internal consistency reliability

All child self-report and parent proxy-report scales exceeded the minimum reliability standard of 0.70 required for group comparisons, with parent proxy-report also suitable for individual analysis (Table 2).

**Table 2:** PedsQL™ internal consistency reliability for child self-report and parent proxy-report for children with severe SLI

Scale	Cronbach’s internal consistency reliability coefficient alpha
Child Self-Report	
Total Summary Score	0.80
Physical Health Summary Score	0.86
Psychosocial Health Summary Score	0.81
Emotional Functioning	0.89
Social Functioning	0.88
School Functioning Summary Score	0.84
Parent Proxy-Report	
Total Summary Score	0.90
Physical Health Summary Score	0.93
Psychosocial Health Summary Score	0.90
Emotional Functioning	0.94
Social Functioning	0.92
School Functioning Summary Score	0.92

### Range of measurement

All child self-report and parent proxy-report scales were below the 15 % level suggested for both ceiling and floor effects (Table 3).

**Table 3:** Percentage of children with severe SLI showing floor and ceiling effects on the PedsQL™

Scale	Floor effect	Ceiling effect
	Presented as percentage	Presented as percentage
Child Self-Report		
Physical Health Functioning	0	7
Emotional Functioning	0	12
Social Functioning	0	12
School Functioning	0	2
Parent Proxy-Report		
Physical Health Functioning	0	7
Emotional Functioning	0	0
Social Functioning	0	5
School Functioning	0	2

### Child self-report and parent proxy-report results

Table 4 presents the results of the PedsQL™ for both the child self-report and parent proxy-report for the children in this study. Group mean scores, along with the percentage of children with impaired scores are shown in Table 4. There were no significant differences between child self-report and parent proxy-report on all scales except social functioning, where children scored themselves significantly better than parents did (*z* = −2.46, *p* = 0.014).

**Table 4:** Descriptive statistics for the PedsQL™ child self-report and parent proxy-report for children with severe SLI

Scale	Range	Mean (SD)	Cut-off scores^b^	Percentage^c^
Child Self-Report				
Total Summary Score	29.35–100	66.51 (15.36)^a^	69.71	60
Physical Health Summary Score	12.50–100	67.00 (22.19)^a^	72.98	49
Psychosocial Health Summary Score	38.33–100	66.24 (14.88)	66.03	59
Emotional Functioning	15–100	68.26 (20.23)	59.57	28
Social Functioning	10–100	66.28 (23.83)^a^	66.61	46
School Functioning Summary Score	30–100	64.19 (16.40)	62.99	52
Parent Proxy-Report				
Total Summary Score	32.61–94.56	63.73 (15.37)^a^	65.42	56
Physical Health Summary Score	15.62–100	69.69 (20.21)	63.28	39
Psychosocial Health Summary Score	33.33–95	60.54 (14.84)^a^	64.38	60
Emotional Functioning	20–95	63.14 (17.99)^a^	63.29	49
Social Functioning	15–100	56.51 (19.13)^a^	62.07	67
School Functioning Summary Score	35–100	61.98 (16.26)	56.75	42

^a^Mean scores that fall below the cut-off mean scores [8]

^b^Mean cut-off scores published in Varni et al. [8]

^c^Percentage of children scoring below the cut-off score for the respective scale

The average age of males was 8.77 years (range 5–15) and of females was 8.88 years (range 5–16). Due to insufficient numbers, the effect of gender and age could not be explored for perceived HRQOL, although the trend is for females to score lower than males. Table 5 presents PedsQL™ gender scale scores, while Table 6 presents PedsQL™ scale scores for each age group where low scores were recorded across all age groups.

**Table 5:** Child-self report of the PedsQL™ for males and females with severe SLI

Scale	Range	Mean (SD)
Males (*n* = 35)		
Total Summary Score	39.13–100	68.01 (15.13)^a^
Physical Health Summary Score	25–100	68.21 (21.75)^a^
Psychosocial Health Summary Score	43.33–100	67.90 (14.77)
Emotional Functioning	40–100	70.71 (18.20)
Social Functioning	10–100	68.57 (22.67)
School Functioning Summary Score	30–100	64.43 (17.27)
Females (*n* = 8)		
Total Summary Score	29.35–82.61	59.92 (15.62)^a^
Physical Health Summary Score	12.5–93.75	61.72 (24.87)^a^
Psychosocial Health Summary Score	38.33–86.67	58.96 (13.91)^a^
Emotional Functioning	15–90	57.50 (26.19)^a^
Social Functioning	10–90	56.25 (27.74)^a^
School Functioning Summary Score	50–80	63.13 (12.80)

^a^Mean scores that fall >1 SD below population mean [8]

**Table 6:** Descriptive statistics for the PedsQL™ child-self report for age groups of children with severe SLI

Scale	Range	Mean (SD)
5–7 year olds (*n* = 21)		
Total Summary Score	39.13–93.48	66.77 (15.19)^a^
Physical Health Summary Score	25–93.75	66.96 (18.87)^a^
Psychosocial Health Summary Score	43.33–96.67	66.66 (15.81)
Emotional Functioning	30–100	70.48 (20.37)
Social Functioning	10–100	64.76 (23.16)^a^
School Functioning Summary Score	30–90	64.76 (17.50)
8–12 year olds (*n* = 18)		
Total Summary Score	29.35–100	66.18 (17.19)^a^
Physical Health Summary Score	12.5–100	64.06 (26.28)^a^
Psychosocial Health Summary Score	38.33–100	67.31 (15.15)
Emotional Functioning	15–100	66.94 (21.43)
Social Functioning	30–100	71.11 (22.53)
School Functioning Summary Score	40–100	63.89 (15.10)
13+ year olds (*n* = 4)		
Total Summary Score	52.17–71.74	66.58 (9.61)^a^
Physical Health Summary Score	56.25–93.75	80.47 (17.75)
Psychosocial Health Summary Score	50–68.33	59.17 (7.51)^a^
Emotional Functioning	50–85	62.50 (16.58)
Social Functioning	10–90	52.50 (33.04)^a^
School Functioning Summary Score	40–80	62.50 (20.62)^a^

^a^Mean scores that fall >1 SD below population mean [8]

## Discussion

The results of this study support the initial feasibility and reliability of using the PedsQL™ child self-report and proxy-report with school-aged children with severe SLI. The fact that there were no missing item responses, suggests that children with severe SLI and their parents are willing and able to provide good-quality data. In addition, the lack of ceiling or floor effects suggests that children with severe SLI were not navigating to either end of the scale. Children with severe SLI and their parents, at no time perceived themselves to score the worst possible score on any item. This was reflected in a consistently 0 % floor effect. This could reflect the fact that the children’s main challenges encompass speech and/or language development which none of the items in this measure specifically target. It can be anticipated that a measure specific for language impairment would produce some floor effects. In addition, failure to produce any significant ceiling effects, suggest that this measure is ideal as it captures challenges children with severe SLI perceive to be difficult across various domains of HRQOL, highlighting the complexity of this developmental disorder. Therefore, it would appear that the PedsQL™ is a promising measure for use with this cohort of children. However, it is worth highlighting that the researchers were dependent on signing the words while reading the questions to most of the children. Without the use of sign language, it is questionable if the children could have answered all the questions. In addition, it is worth noting that the simplicity of the vocabulary that Varni et al. (1999) have used in developing this scale facilitated both the comprehension of questions by the child with language impairment (in the absence of cognitive impairment), and the researchers’ capacity to precisely sign the sentence to avoid any bias. Although the measure is quick to administer, some children became distracted and appeared to focus less on the work at hand. When this occurred, the child was given a break to complete some movement tasks, such as jumping activities. One small break seemed to be enough to redirect the child’s attention to the questionnaire allowing for completion. Future researchers may need to adopt the above-mentioned strategies in order to optimize data collection from children with severe SLI.

Consistent with previous literature [11], this study confirmed the hypothesis that children with severe SLI and their parents both perceived the child to be at-risk for impaired overall HRQOL relative to the healthy population sample, reflective of scores obtained by children with physician-diagnosed severe chronic health conditions [8]. The total HRQOL summary score fell below the cut-off score for 60 % of children with severe SLI, with a similar 56 % of parents rating their children below the cut-off score.

It has been reported that parents more often score their children worse than their child on measures of HRQOL [26]. Consistent with this literature, the parents of children with severe SLI rated their child’s HRQOL worse than their children themselves across all domains except physical functioning [26]. However, only the social functioning domain reached significance, with parents (similar to their children) reporting low social functioning but at a significantly lower rate. It has been reported that the primary caregiver is the most valid proxy [24]. In this study, 100 % of mothers, of whom a large percentage were stay at home mothers, completed the questionnaire. Considering children and parents did not complete their questionnaire simultaneously, it would appear that the responses from the children seem reasonable in comparison to the mother’s. While the authors are not suggesting a proxy-report is entirely adequate to replace the child’s report, the marked similarities between parent and child reports suggest that these children can reflect and provide information pertaining to their own HRQOL.

Further investigation into the child and parent responses reveals that out of the four scale scores, children rated both physical and social functioning below the cut-off point. Interestingly, contrary to our hypothesis, the children in this study rated physical functioning the lowest. It may be expected that a group of children with severe communication challenges would identify their own poor social functioning. However, it may not have been anticipated that these children would rate their physical functioning low as well, considering their most evident challenge is communication, and not physical. However, this outcome complements emerging evidence that up to 90 % of children with various speech-language delays/disorders present with motor comorbidity to some extent [4]. Until now it was not clear that the motor comorbidity also impacted their HRQOL, and that together the perceived impairment in social and motor performance was severe enough to impact the total summary score for 60 % of the children in this study. These results contribute to the on-going debate within the literature supporting the premise that SLI is not limited to language impairments, but encompass problems in other developmental domains. Parents did not perceive an impact on physical functioning to the same extent as their children. In fact, compared to the cut-off scores, parents’ perceptions of physical functioning had the largest difference, however, unlike the children the parents mean score fell above the healthy population cut-off score. This difference may be because parents see their child’s language impairment as the primary impairment, and therefore may not identify physical challenges as a problem. This is consistent with the literature where parents often overestimated their child’s gross motor skills in children with vocabulary difficulties [27, 28]. This is concerning as it is parents’ unique perceptions of their child’s HRQOL that guide and direct clinical decision making and utilization of health care services [17, 29, 30]. Parents failing to identify their child’s physical challenges will likely not seek services to improve physical capacity, potentially missing opportunistic intervention. Although the parents did not perceive physical functioning problems to the extent that their child did, the results from this study suggest that HRQOL measures used with children with severe SLI should embrace questions pertaining to physical functioning if the researchers or clinicians wish to gain an understanding of the extent of difficulties perceived by the child and his/her parent. Further, this will ensure children are referred to inter-professional management accordingly, to enhance management. Unlike the children, out of the four scale scores, parents reported both emotional and social functioning below the cut-off score. For emotional functioning, 50 % of parents scored their child below the cut-off score, with the mean score only just falling below the at-risk score. In comparison only 28 % of children rated themselves below the cut-off score. Although the pre-defined 15 % threshold was not met, 12 % of children had a ceiling effect in emotional functioning suggesting these children perceived themselves as not feeling scared, sad, angry, and worried or as having troubles sleeping. It could equally be argued that the children drifted towards the ceiling because they had difficulties understanding these emotions, however, the responses were not consistent enough to create a significant ceiling effect, so this measure remains useful for this cohort. A significant difference in the social functioning scores may reflect the parents’ increased awareness of disparities and broader basis for comparison of their child’s functioning, while the children may have rated this based on their main socializing experiences amongst like individuals, which was at the dedicated school. Similarly, the pre-defined 15 % threshold was not met, yet 12 % of children had a ceiling effect in social functioning supporting the premise that children perceived themselves able to get along with other children and make friends, likely within the protected environment of the school.

Considering the children in this study are schooled in a setting catering to children with similar diagnoses, it is understandable that both children and their parents rated school functioning above the cut-off score. However, the child school functioning mean score was just above the cut-off score, and 42 % of parents along with 51 % of children, perceived the child’s school functioning to be “at-risk.” This suggests that even in a protected setting, many children with severe SLI and their parents identify difficulties in school functioning.

Although females consistently scored themselves lower than males, caution is needed when interpreting this due to the small numbers of females impacting data analysis. However, the large percentage of males in our study reflects the higher prevalence of males diagnosed with SLI [1]. Similarly, there were differences between the age groups, however, limited numbers impacted data analysis. Importantly, the age group results suggest that children with severe SLI do not grow out of their impairment and continue to perceive an impact on their HRQOL well into adolescence. Scale score differences between the age groups likely reflect the perceptions of importance that different domains have at different ages. For example, younger children who often engage in more movement-based play perceived their physical functioning to be “at-risk” for impairment, whereas, older children who likely spend more time socializing and studying identified social and school difficulties. Further, any real differences could be due to an increased awareness of disparities between themselves and typically developing peers due to unique life experiences. Consequently, further research is required to explore the HRQOL of children with SLI from both larger samples, longitudinally, and across subgroups not only age and gender, but also receptive compared to expressive language impairments. In order to further guide the management of children with SLI, studies should qualitatively explore reasons for disparities, or analyze assessment findings in light of HRQOL perspectives. This study has confirmed the suitability of the PedsQL™ for use with children with severe SLI, yet there may be another HRQOL measure that is equally suitable or even optimal for use with this cohort. Gomersall et al. [15] proposed the comparison of multiple generic HRQOL measures in one study. The PedsQL™ compared to other measures will be an ideal approach to identifying the optimal measure for clinicians to use with children with severe SLI.

Limitations of this study include data collection from one school, with a small sample size, particularly the number of females and older children limiting analysis. However, this study is the first to explore the HRQOL of children with severe SLI and provides preliminary evidence that SLI impacts multiple HRQOL domains and that these children should not be excluded from future studies.

## Conclusions

In conclusion, the PedsQL™ was a suitable measure that identified children with severe SLI to be at risk of lower HRQOL. Children with severe SLI should be given the opportunity to express their own perceptions, before resorting to a proxy-report. However, the value of additional information obtained from a proxy-report advocate for its completion as well. Health professionals working with children who have SLI need to consider not only a child’s impairment, but also their wellbeing and participation by incorporating HRQOL measures into assessment. This will empower children while assisting in clinical decision making to ensure therapeutic outcomes are meaningful and impact positively on a child’s life.

## Abbreviations

HRQOL: Health-related Quality of Life
PedsQL™: Pediatric Quality of Life Inventory™ 4.0 Generic Core Scales
SLI: Specific language impairment
SD: Standard deviations
DETE: The Department of Education, Training and Employment
